# Combined sunitinib and radiation therapy for preoperative treatment of soft tissue sarcoma: results of a phase I trial of the German interdisciplinary sarcoma group (GISG-03)

**DOI:** 10.1186/s13014-016-0654-2

**Published:** 2016-06-03

**Authors:** Jens Jakob, Anna Simeonova, Bernd Kasper, Ulrich Ronellenfitsch, Geraldine Rauch, Frederik Wenz, Peter Hohenberger

**Affiliations:** Division of Surgical, Oncology and Thoracic Surgery, Department of Surgery, University Medical Center and Medical Faculty Mannheim, University of Heidelberg, Th.-Kutzer-Ufer 1-3, Mannheim, 68137 Germany; Department of Radiation Oncology, University Medical Center Mannheim, University of Heidelberg, Th.-Kutzer-ufer 1-3, Mannheim, 68137 Germany; Sarcoma Unit, Interdisciplinary Tumor Center Mannheim, Mannheim University Medical Center, University of Heidelberg, Th.-Kutzer-Ufer 1-3, Mannheim, 68137 Germany; Division of Surgical, Oncology and Thoracic Surgery, Department of Surgery, University Medical Center Mannheim, University of Heidelberg, Th.-Kutzer-Ufer 1-3, Mannheim, 68137 Germany; Institute of Medical Biometry and Informatics, University of Heidelberg, ImNeuenheimer Feld 305, Heidelberg, Germany; Department of Radiation Oncology, University Medical Center and Medical Faculty Mannheim, University of Heidelberg, Th.-Kutzer-Ufer 1-3, Mannheim, 68137 Germany

## Abstract

**Background:**

Experimental data demonstrated that concurrent anti-angiogenic treatment with sunitinib may improve the efficacy of radiation therapy (RT). Here we report the results of a phase I trial performed within the German Interdisciplinary Sarcoma Group (GISG-03) of combined sunitinib and RT for neoadjuvant treatment of locally advanced soft tissue sarcoma (STS).

**Methods:**

The primary endpoint of the study was to explore the recommended dose of sunitinib combined with RT for subsequent trials. Treatment response, postoperative complications after tumor resection and toxicity according to CTCAE 4.0 were secondary endpoints. The study used a 3 + 3 design. Patients received either 25 mg (dose level 1) or 37.5 mg (dose level 2) sunitinib two weeks prior to and throughout RT (28 × 1.8 Gy). Surgery was scheduled 5–8 weeks after completion of neoadjuvant treatment. Study registration: NCT01498835.

**Results:**

Six patients were enrolled in dose level 1 and three patients in dose level 2. Median tumor size was 11 cm. Tumors were located in the retroperitoneum (4/9), lower leg (3/9) or trunk (2/9). At dose level 1, 1/6 patients developed dose limiting lymphopenia. At dose level 2, no patient developed dose limiting toxicity. Most frequent toxicities were hematological (8/9) and oral (5/9). Dose adjustments of sunitinib were necessary in 5/9 patients. All patients received full dose RT and underwent tumor resection (8/9 R0 and 1/9 R1). Local toxicity of RT did not exceed Grade 2. 2/9 patients had postoperative complications requiring re-intervention. Treatment response according to RECIST was as follows: partial response 1/9, stable disease 7/9, and progressive disease 1/9. Pathological examination revealed ≥ 95 % tumor necrosis in 3/9 resected specimens.

**Conclusions:**

Combined sunitinib and RT was tolerable as neoadjuvant treatment for locally advanced STS patients regardless of tumor localization. The recommended sunitinib dose for subsequent trials is 37.5 mg.

## Introduction

Soft tissue sarcomas (STS) are a rare group of mesenchymal tumors comprising approximately 1 % of all malignancies [1]. Surgical resection and pre- or postoperative irradiation is the standard treatment for locally advanced STS of the extremities [2, 3]. In retroperitoneal STS preoperative radiation therapy (RT) is currently under investigation in a phase III trial after cohort studies had demonstrated that preoperative RT may decrease local recurrence and improve overall survival [4, 5]. (EORTC STRASS trial ClinicalTrials.gov Identifier: NCT01344018) Despite improvements in irradiation techniques such as intensity modulated radiation therapy (IMRT) local recurrence rates of large, high grade STS is still up to 20 % in extremity and 40 % in retroperitoneal tumors and there is a definitive need to improve local therapy [6, 7].

Receptor tyrosine kinase inhibitors such as sunitinib exhibit antiproliferative and anti-angiogenic properties [8]. Experimental data demonstrated that concurrent treatment with sunitinib and RT is more effective than RT alone in engineered mouse models of soft tissue sarcoma [9]. Thus, concurrent treatment seems to lead to additive effects. Jain et al. postulated even synergistic effects if anti-angiogenic treatment is administered upfront RT by “normalizing” the chaotic neovascularization of tumors [10]. Vascular normalization may then lead to decreased tumor hypoxia and consequently to increased RT efficacy.

Here we report the results of a phase I trial of concurrent sunitinib and RT in patients with locally advanced soft tissue sarcoma conducted within the German Interdisciplinary Sarcoma Group (GISG). The rationale and the design of the trial have been described in detail before [11].

## Methods

### Study design

This was an open-label, single-arm, single center phase I trial performed within the GISG. The study intervention was administration of sunitinib concurrently to preoperative irradiation in patients with soft tissue sarcoma. The primary endpoint of the study was to determine the recommended phase II dose of sunitinib given two weeks before and throughout standard RT as preoperative treatment. Secondary endpoints were toxicity, radiographic and pathologic treatment response as well as morbidity of tumor resection. A standard dose escalation 3 + 3 design with two dose levels was used.

The sponsor of the trial was the University of Heidelberg, Germany. The study was funded by the German Research Foundation (DFG, grant number JA 2030/1) and the Medical Faculty Mannheim, University of Heidelberg, Germany. The study medication was provided by Pfizer Oncology Germany. Pfizer had no influence on design, conduction or publication of the trial. The study was approved by the Ethics Committee II of the University of Heidelberg and the Federal Agency for Drugs and Medical Devices, Germany (BfArM). The trial was registered at clinicaltrials.gov (NCT01498835). The trial was performed on an outpatient basis. All patients were treated at the Mannheim University Medical Center (UMM), University of Heidelberg, Germany.

### Patient selection

Patients with locally advanced primary soft tissue sarcoma that required preoperative irradiation and tumor resection were included into the trial. The protocol allowed recruitment of patients with primary tumors but also with local recurrences or metachronous solitary metastatic lesions if the treatment decision was to perform RT and subsequent surgery. All tumors had to be resectable and biopsy-proven STS. Patients needed to have an age of 18 years or older with ECOG status 0 or 1 and normal organ function (left ventricular ejection fraction, kidney, liver, and bone marrow). Main exclusion criteria were metastatic disease (with the above mentioned exception), prior therapy with receptor tyrosine kinase inhibitors or conventional chemotherapy or history of myocardial infarction, congestive heart failure, stroke, thrombosis or embolism or uncontrolled medical disease such as arterial hypertension or diabetes mellitus.

### Treatment regimen

The treatment plan was to administer sunitinib two weeks prior to and during intensity modulated radiation therapy (IMRT, 28 × 1.8 Gy) and to resect the tumors 5 to 8 weeks after the end of the neoadjuvant treatment. Adjuvant chemotherapy was not administered. All procedures were described in detail before [11].

Sunitinib malate was orally administered as hard gelatin capsules of 25 or 12.5 mg. Sunitinib dose was escalated according to a traditional 3 + 3 design. Dose level 1 was 25 mg and dose level 2 was 37.5 mg. No further dose escalation was planned. Intrapatient dose escalation was not permitted. Sunitinib was taken starting 2 weeks prior to radiation therapy, continued throughout radiation therapy and stopped on the final day of radiation therapy. Sunitinib was administered continuously and not with a 4 weeks on/2 weeks off regimen.

Intensity-modulated radiation therapy (IMRT) was applied according to standard protocols at the Mannheim University Medical Center. The dose of IMRT was 50.4 Gy (median planning target volume (PTV) dose) administered in 28 fractions within 5.5 weeks. Dose prescription was performed at the median dose in the PTV with the 90 % isodose line covering the PTV. A CT simulation was performed to define the gross tumour volume (GTV). In case of retroperitoneal sarcomas, the clinical target volume (CTV) was the GTV with a 1,5 cm margin axially. The margin placed around the GTV in the superior-inferior direction was 2,5 cm. The planning target volume was the CTV with a 5 mm margin. In case of extremity STS the CTV margins were 2,5 cm axially and 4,5 cm in the superior-inferior direction. The dose constraint for the small intestine was 45 Gy at the maximum and the mean dose for kidneys were not greater than 10 Gy. Treatment was performed using step-and-shoot IMRT. A strip of 2–3 cm skin was spared in case of extremity STS.

Surgery was scheduled 5–8 weeks after completion of neoadjuvant treatment. Surgery was planned as compartment or wide resection in extremity STS. In retroperitoneal sarcoma, the surgical approach was a multivisceral resection including adjacent organs (e.g. colectomy, nephrectomy, splenectomy, distal pancreatectomy) and resection of abdominal wall musculature or, respectively, the psoasmuscle.

### Dose limiting toxicity (DLT) and dose adjustments of sunitinib

Adverse events occurring during intake of sunitinib until 4 weeks after completion of combined RT and sunitinib were documented and classified according to common toxicity criteria of adverse events (CTCAE 4.0) by weekly study visits including clinical examination, interrogation for adverse events, laboratory tests and ECG. DLTs were defined as any toxicity that causes discontinuation of RT for more than 6 days, toxicity classified grade 4 or 5 and toxicity classified grade 3 with the exception of hematologic toxicities, arterial hypertension if controlled by adequate medication within 14 days and elevated serum levels of liver and pancreas enzymes if not related to clinical symptoms. Sunitinib was stopped and not reintroduced in case of DLT. In case of grade 3 hematological toxicity sunitinib was reduced (37.5 mg to 25 mg to 12.5 mg daily) until recovery to grade 1 and then reintroduced in the original dose. Furthermore the protocol allowed a temporary dose reduction in case of prolonged toxicity of any CTCAE grade on an individual basis. All dose reductions were documented in detail. Postoperative morbidity was assessed by a study visit between the 30^th^ and 40^th^ day after tumor resection and also classified according to CTCAE 4.0.

### Evaluation of treatment response

Imaging response was defined according to response evaluation criteria in solid tumors (RECIST) by comparing pre-treatment and preoperative magnetic resonance imaging or computed tomography. Pathological response was defined as the fraction of non-viable tumor tissue in the resection specimen with cut-off points at fifty and ninety per cent.

## Results

### Patients and tumor characteristics

A total of 10 patients were recruited. The cohort of dose level 1 was extended from three to six patients as planned in the study protocol since one of the first three patients suffered dose limiting toxicity. Three patients were treated at dose level 2 as planned in the study protocol. One patient was recruited into the study but actually did not meet all inclusion criteria after pathology review. Therefore this patient had to be excluded from the analysis. The patient never started study treatment and was treated by surgery alone. All following analyses are based on the nine patients treated according to the study protocol. Patient characteristics are depicted in Table 1.

**Table 1:** Patient and tumor characteristics

Number of patients	9
Dose level 1 (25 mg sunitinib)	6
Dose level 2 (37.5 mg sunitinib)	3
Gender	
Female	3
Male	6
Age	
Median (range), years	52 (29–75)
Tumor site	
Retroperitoneum	4
Lower Extremity	3
Groin	1
Chest wall	1
Tumor size	
Median (range), cm	11 (5–20)
Grading (FNLCC)	
Low grade	1
Intermediate grade	1
High grade	7
Histology	
Dedifferentiated liposarcoma	4
Undifferentiated pleomorphic sarcoma	2
Myxoid liposarcoma	1
Myxofibrosarcoma	1
Synovial sarcoma	1

### Toxicity of preoperative treatment

At dose level 1, 1/3 initially recruited patients developed a DLT (grade 4 lymphopenia) but recovered completely after discontinuation of sunitinib. This patient had a retroperitoneal dedifferentiated liposarcoma with a size of 15 cm. The dose level 1 cohort was then expanded to six patients. None of the following dose level 1 patients developed DLT. At dose level 2, 0/3 patients developed DLT.

The most frequent toxicity of any grade (8/9) and most frequent grade 3 or 4 toxicity (5/9) was hematotoxicity. Hematotoxicity resolved in all patients before surgery and no patient developed neutropenic fever. Grade 3/4 hematotoxicity occurred at both dose levels and in patients with tumors located in the retroperitoneum as well as located in the extremities. No other grade 3 toxicity occurred. Hematotoxicity was the most frequent toxicity of any grade. Six out of nine patients required dose adjustments due to toxicity. Five patients had dose adjustments because of hematotoxicity and one patient because of prolonged grade 2 mucositis. Dose adjustments were necessary at both dose levels. In 5/6 patients with dose adjustments, dose reductions were necessary after 4 or more weeks of treatment with sunitinib. Radiation therapy was completed without toxicity related dose reductions or delays in all patients. Toxicity within the radiation field did not exceed grade 2 in any patient. One patient had a break of radiation therapy for one day due to gastrointestinal toxicity. All other patients completed radiation therapy as planned. Toxicity of combined treatment is summarized in Table 2.

**Table 2:** Toxicity of study treatment and postoperative complications of the individual patients

Dose level	Patient	Tumor site	Toxicity of combined sunitinib and RT (CTCAE 4.0 grade)	Radiation site toxicity (CTCAE 4.0 grade)	Postoperative complications (CTCAE 4.0 grade)
1	1	Lower extremity	neutropenia (3) thrombocytopenia (2) facial edema (1) fatigue (1)	0	prolonged wound drainage (1)
1	2	Lower extremity	-	0	wound dehiscence (3)
1	3	Retroperitoneal	lymphopenia (4) neutropenia (3) thrombocytopenia (3) gamma-glutamyl-transferase elevation (2) arterial hypertension (2) fatigue (1)	1	-
1	4	Retroperitoneal	lymphopenia (3) neutropenia (2) thrombocytopenia (1) genitourinary (2) diarrhea (2)	0	intraoperative bleeding (4) intraabd. abscess (3) myocardial ischemia (3) anastomotic leakage (3)
1	5	Retroperitoneal	lymphopenia (3) neutropenia (2) thrombocytopenia (1) arterial hypertension (2) dry mouth (1) diarrhea (1)	1	-
1	6	Groin	lymphocytopenia (3) neutropenia (1) diarrhea (2) dysgeusia (1)	2	-
2	7	Retroperitoneal	lymphopenia (2) neutropenia (2) thrombocytopenia (1) nausea (2) dry mouth (2)	2	-
2	8	Lower extremity	neutropenia (1) thrombocytopenia (1) vaginal mucositis (2) hand foot syndrome (1) nausea (1) oral mucositis (1) epistaxis (1)	2	prolonged wound drainage (2) lymphedema (2)
2	9	Chest wall	lymphopenia (3) neutropenia (1) thrombocytopenia (1) oral mucositis (1) fatigue (1)	1	delayed wound healing (1)

### Surgery and postoperative morbidity

All patients underwent tumor resection within 4 to 10 (median 7) weeks after completion of preoperative treatment. The four patients with extremity STS underwent wide resection, one required reconstruction of the superficial femoral artery and one required plastic reconstruction with a gastrocnemius flap. Three out of four patients with retroperitoneal sarcoma underwent multivisceral resection with colectomy and nephrectomy, one patient with a retroperitoneal undifferentiated sarcoma was diagnosed with several small size liver metastases at surgery and underwent tumor resection with colectomy and without nephrectomy. One patient had a chest wall sarcoma and underwent tumor resection en bloc with chest wall and upper lobe resection. Microscopic clear margins for the primary tumor site were achieved in 7/9 patients and 2/9 patients had R1 (microscopically incomplete) resections. Five out of nine patients developed postoperative complications and 2/9 patients required re-interventions (Table 2). One patient with extremity STS went back to theatre because of wound dehiscence and one patient with a retroperitoneal sarcoma had intraoperative bleeding, postoperative anastomotic leakage and abscess formation. There was no obvious difference in postoperative morbidity regarding dose level (3/5 dose level 1 vs. 2/5 dose level 2, *p* = 0.60) or sunitinib dose reductions (4/5 with dose reduction, 1/5 without dose reduction, *p* = 0.40).

### Treatment response and oncological outcome

Restaging before surgery showed partial response in one patient, stable disease in seven patients, and progressive disease in one patient (Table 3). The patient with progressive disease had a retroperitoneal undifferentiated pleomorphic sarcoma with progression of the primary tumor and two liver metastases at surgical exploration. Pathological examination revealed ≥ 95 % non-viable tissue in 3/9 resection specimens (Table 3). Histology of these patients was dedifferentiated liposarcoma, synovial sarcoma and undifferentiated pleomorphic sarcoma.

**Table 3:** Treatment response and oncological outcome

Patient	Tumor site, histology and size	Sunitinib dose	RT dose	Resection margin	RECIST	% non-viable tissue	Follow-up status (and time), events (months after ED)
1	lower extremity, myxofibrosarcoma, 11 cm	25	50.4	R0	stable disease	30 %	NED (29mo), LR resected (19 mo)
2	lower extremity, dediff. lipo, 8 cm	25	50.4	R0	stable disease	95 %	AWD (36 mo), Soft tissue mets (15 mo)
3	Retroperitoneal, dediff. lipo, 15 cm	25	50.4	R1	stable disease	75 %	NED (28 mo)
4	Retroperitoneal, dediff. lipo, 12 cm	25	50.4	R1	stable disease	20 %	NED (11 mo)
5	Retroperitoneal, undiff. pleomorphic sarcoma, 14 cm	25	50.4	R2 (primary Tumor R0)	progressive disease (locally and mets)	70 %	DOD (9 mo), Liver mets at surgery
6	Groin, Dediff. lipo, 20 cm	25	50.4	R0	stable disease	30 %	NED (24 mo)
7	Retroperitoneal, myxoidlipo, 5 cm	37.5	50.4	R0	partial response	50 %	NED (20 mo)
8	lower extremity, synovial sarcoma, 8 cm	37.5	50.4	R0	stabledisease	100 %	AWD (23 mo), Lung mets (15 mo)
9	chest wall, undiff. pleomorphic sarcoma, 5 cm	37.5	50.4	R0	stabledisease	100 %	NED (13 mo)

Median follow-up was 23 months (range 9–36 months, Table 3). One patient developed a local recurrence of a myxofibrosarcoma of the thigh within the radiation field after 19 months. Three patients were diagnosed with metastatic disease. One patient with metastatic disease died during follow-up.

## Discussion

The primary endpoint of this GISG phase I trial was to determine the recommended dose of sunitinib given concurrently with preoperative RT in locally advanced soft tissue sarcoma patients. The most relevant toxicity of combined RT and sunitinib was hematotoxicity. Eight out of nine patients developed hematotoxicity, four out of nine patients hematotoxicity grade 3 (CTCAE 4.0) and one patient had a dose limiting grade 4 lymphopenia. According to the 3 + 3 design of the trial, the recommended dose of sunitinib for further trials is 37.5 mg given daily and continuously per os starting two weeks prior to the first day and until the last day of RT.

Combined anti-angiogenic treatment was evaluated in several other trials: Pazopanib, sorafenib, sunitinib and bevacizumab were administered concurrently with RT to determine toxicity of combined treatment as a primary endpoint and to explore its efficacy (Table 4) [12–17]. All but one study demonstrated that the combination of RT with anti-angiogenic drugs does not result in excessive toxicity, does not interfere with a full dose administration of RT and is not associated with higher than expected postoperative morbidity. The toxicity profile of combined treatment reflects the toxicity profile of the administered anti-angiogenic drug(e.g. hepatotoxicity in case of pazopanib or hematotoxicity in case of sunitinib) meaning that the combination of the anti-angiogenic drug with radiation therapy did not lead to unexpected adverse events. It is remarkable though, that in the trials the proportions of patients who suffered from grade 3 toxicities were generally higher in the combination with RT compared to anti-angiogenic monotherapy (e.g. hepatotoxicity in case of pazopanib or hematotoxicity in case of sunitinib) [18, 19]. The increased grade of known and typical toxicities may be attributed to the combination with RT. It is well known that the RT dose administered to the bone marrow has a relevant and overproportional influence on the occurrence and severity of hematotoxicity when combined with chemotherapy as compared to RT alone [20, 21]. The same appears to be true for concurrent treatment with anti-angiogenic agents. Yet, there is no obvious explanation for increased hepatotoxicity of combined treatment compared to anti-angiogenic drug monotherapy since hepatotoxicity was also increased in patients with extremity tumors with no radiation dose to the liver [15]. Lewin et al. published the only trial that resulted in unacceptable toxicity with severe hepatotoxicity and hematotoxicity when RT was combined with sunitinib in extremity STS [13]. Their results contradict our own phase I trial and the results of Sunyach et al. who presented their data on combined RT and sunitinib in irresectable extremity STS during the conference of the connective tissue oncology society 2014 [16]. In principal, the three trials used similar treatment regimens. Yet, Lewin et al. administered a higher starting dose (50 mg for two weeks followed by 25 mg thereafter) which may have resulted in higher toxicity.

**Table 4:** Trials evaluating preoperative anti-angiogenic treatment and RT in STS

Author	Evaluated patients	Treatment regimen	Tumor site	Patients with toxicity (CTCAE) ≥ grade 3	Type of toxicity (CTCAE) ≥ grade 3	Path. response: ≥ 90 % non-viable tissue
Haas 2015	11	Pazopanib/RT	Extremity (*n* = 11)	3/11	Hepatotoxicity	4/10
Canter 2014	8	Sorafenib/RT	Extremity (*n* = 8)	4/8	Skin rash (*n* = 2), anemia, perirectal abscess, supraventricular tachycardia	3/8
Yoon 2011	20	Bevacizumab/RT	Extremity (*n* = 13) Retro (*n* = 6) Trunk (*n* = 1)	4/20	Hepatotoxicity, hypertension	7/20
Lewin 2014	9	Sunitinib/RT	Extremity (*n* = 9)	7/9	Hepatotoxicity, hematotoxicity, hyponatremia, hyperglycemia, skin rash	Not reported
Jakob 2015	16	Sunitinib/RT	Extremity (*n* = 5) Retro (*n* = 10) Trunk (*n* = 1)	8/16	Hematotoxicity, hand-foot-syndrome	5/16
Sunrase (this trial)	9	Sunitinib/RT	Extremity (*n* = 3) Retro (*n* = 4) Trunk (*n* = 2)	5/9	Hematotoxicity	3/9
Sunyach 2014	10	Sunitinib/RT	Extremity (*n* = 10)	Only DLT reported	Thrombopenia grade 4 (*n* = 1), no other dose limiting toxicity	Not applicable, therapy given for unresectable tumors

The aim of combining anti-angiogenic drugs and RT is to increase RT efficacy and to improve local tumor control. None of the above mentioned trials was designed as a phase II or III trial. Nevertheless, most of the authors presented data on pathological response as a surrogate marker of treatment efficacy (Table 4). Although the evaluation of pathologic response has not been standardized until recently and the fraction of non-viable tumor in the resection specimen depends on histological subtype, it may be used to estimate the efficacy of combined modality treatment in general [22]. Noteworthy, more than a third of the treated patients had tumors with less than 10 % viable tumor cells in all five trials that reported pathologic response (Table 4). This proportion appears to be significantly higher than what would be expected after preoperative RT alone [23]. This implies that RT and anti-angiogenic drugs indeed have additive effects. Serum biomarkers of angiogenesis and functional imaging may be alternative surrogate markers for treatment response. Yoon et al. and Lewin et al. performed functional imaging studies after the run-in phase of bevacizumab or sunitinib and after combined treatment, respectively [13, 17]. Both groups applied different imaging techniques (perfusion CT vs. PET-CT and functional MRI) and found heterogeneous results after anti-angiogenic monotherapy (no influence on median blood flow vs. increased hypoxia, respectively) whereas combined treatment led to decreased blood flow and tumor hypoxia, respectively. Canter et al. evaluated the course of the angiogenesis markers VEGF, EGF and PDGF during neoadjuvant treatment [14]. Overall, there was no significant difference in serum levels between treatment responders and non-responders. These results demonstrate the difficulty to define reliable predictors of response to combined RT and anti-angiogenic treatment. With PAZNTIS, a phase II/III trial has already been initiated by the American Children’s Oncology Group that is sponsored by the NCI. The trial will test preoperative RT vs. pazopanib/RT vs. chemotherapy/RT vs. pazopanib/chemotherapy/RT (ClinicalTrials.gov Identifier: NCT02180867). The PAZNTIS trial will use pathologic response with a cut off at 90 %, serial PET-CT imaging and circulating DNA as surrogate markers of response. Pediatric as well as adult patients may be included with a number of restrictions in the eligibility criteria for recruitment into the different treatment arms. These restrictions and the fact that this is a four arm study with pediatric and adult patients will render results difficult to interpret. Yet, no other phase II/III study of combined treatment has been initiated so far.

## Conclusions

The combination of anti-angiogenic drugs and RT is feasible and toxicity of combined treatment is tolerable. Surgery appears to be safe after concurrent treatment regimens. Further trials need to take into account histological subtypes. Surrogate markers of tumor response are necessary to define the efficacy of neoadjuvant combined RT and anti-angiogenic drug treatment in STS.

## Abbreviations

CT, computed tomography: CTCAE, common terminology criteria for adverse events: CTV, clinical target volume: DLT, dose limiting toxicity: ECG, electrocardiogram: ECOG, Eastern Cooperative Oncology Group: GISG, german interdisciplinary study group: GTV, gross tumour volume: Gy, Gray: IMRT, intensity modulated radiation therapy: PET, positron emission tomography: PTV, planning target volume: RECIST, response evaluation criteria in solid tumors: RT, radiation therapy: STS, soft tissue sarcoma
